# The DSM-5: Hyperbole, Hope or Hypothesis?

**DOI:** 10.1186/1741-7015-11-128

**Published:** 2013-05-14

**Authors:** Michael Berk

**Affiliations:** 1 School of Medicine, Deakin University, Barwon Health, Ryrie Street, Geelong, VIC 3220, Australia; 2 Department of Psychiatry, Orygen Research Centre, and the Florey Institute for Neuroscience and Mental Health, The University of Melbourne, Parkville, VIC 3052, Australia

**Keywords:** DSM-V, Diagnosis, Pathophysiology, DSM-V, Symptoms, Classification

## Abstract

The furore preceding the release of the new edition of the Diagnostic and Statistical Manual of Mental Disorders (DSM-5) is in contrast to the incremental changes to several diagnostic categories, which are derived from new research since its predecessor’s birth in 1990. While many of these changes are indeed controversial, they do reflect the intrinsic ambiguity of the extant literature. Additionally, this may be a mirror of the frustration of the field’s limited progress, especially given the false hopes at the dawn of the “decade of the brain”. In the absence of a coherent pathophysiology, the DSM remains no more than a set of consensus based operationalized adjectives, albeit with some degree of reliability. It does not cleave nature at its joints, nor does it aim to, but neither does alternate systems. The largest problem with the DSM system is how it’s used; sometimes too loosely by clinicians, and too rigidly by regulators, insurers, lawyers and at times researchers, who afford it reference and deference disproportionate to its overt acknowledged limitations.

## 

*“Essentially, all models are wrong, but some are useful.”* GEP Box [1]

## Editorial

There has been collective flurry of introspection, debate and controversy about the impact and relevance and criteria for the diagnoses proposed in the upcoming *Diagnostic and Statistical Manual of Mental Disorders* (DSM), Fifth Edition (DSM-5). At least part of the issue is the prevailing zeitgeist; the launch of the DSM-III over three decades ago was paralleled by a retrospectively unrealistic enthusiasm for the manual, its validity and the potential to haul a psychoanalytically oriented discipline into a scientific era. The fundamental problem however remains; the DSM is a symptom based classification, and there remains no coherent pathophysiological foundation for the discipline, the edifice on which medical nosology traditionally is built. It is unrealistic to expect phenomenology to track pathophysiology; nowhere in the rest of medicine does this occur. Cough, pyrexia or pain are all pleomorphic manifestations of diverse pathologies, and depression, anxiety and psychosis are unlikely to be different. The issue is expecting it to be so, as the fundamental validity of the system is clearly absent, but equally obviously, no valid alternate system is in sight.

The climate has thus swung to a wintery disillusionment regarding the perceived failures of the system, with little enthusiasm for the changes, and in particular the expansion of the number and subtypes of diagnostic categories. An analogy is color; using the analogy that we have no idea of the physics of light, that the construct of wavelength even exists, let alone determines color, is it useful to replace “blue” with a series of subtypes of “blue” turquoise, aquamarine, azure, etc.? In this regard, the expanded DSM-5 categorisation represents a greatly expanded series of adjectives or metaphors, able to describe what we see in a manner that is defined with some reliability, even if it fails to explain why blue really is blue.

Biomarker research has largely not supported this current nosology, with markers of cognition, imaging, genetics, inflammation, oxidative stress and neurotrophins showing remarkable homology across categories. Categories including psychotic, mood, personality disorders, and anxiety disorders are associated with common etiological factors including early childhood experiences, social stressors, trauma, personality styles, interpersonal, family, lifestyle, medical, with each factor playing a differential role for each person. Lastly, with the possible exception of lithium, where lithium responsivity does seem to parse a clinically meaningful subgroup, almost all psychotropics show wanton disregard for diagnostic categories in their response profiles, with the atypical antipsychotics an exemplar.

Perhaps the major problem with the DSM system is not the system itself, but how it is used. Firstly, clinicians rarely use these categories rigidly, rather using them as best-fit, pattern-recognition adjectives. Furthermore, there is a tendency for clinicians to focus on axis 1 diagnoses, and while the 5 axis system aims to capture additional domains, it is not a substitute for a comprehensive clinical formulation incorporating developmental history, attachment style, cultural context, defence mechanisms, current life context or the person’s experience of their disorder [2,3]. Phenomenology, as defined by the DSM-5 axis 1, represents the pathophysiological fault line cracked open when the moulded wedge of perception, personality, health and development are struck by the mallet of life. Adjunctive use of formulation adds to understanding which significant factors may have influenced the person’s presentation; identifying key obstacles; suggesting which interventions might be more useful and anticipating which challenges are likely to occur during the course of management. Secondly, regulators, insurers and the legal system frequently take DSM categories at face value, and imbue them with a level of deference that is discordant with their underlying validity, setting structures in place that clinicians are obliged to follow despite knowing intrinsically that they are incorrect. The controversy regarding on versus off-label prescription would be perhaps the exemplar.

All systems reflect the cultural system in which they evolve, and the DSM-5 is no exception, being tailored to a significant degree to the insurance, regulatory and reimbursement systems in play in the USA. The 10th edition of the International Classification of Diseases (ICD-10), it’s not too distant sibling, arguably reflects a more global set of inputs and might be more universally translatable, although it needs to be emphasised that the similarities between the systems are far greater than the differences, and the ICD solves none of the primary issues that bedevil the DSM-5. Lastly, and perhaps most seriously, using DSM categories as railway tracks for research risks limiting the exploration of novel or more promising nosologies or dichotomies.

So where does this leave us? The DSM-5 is a bit like Churchill’s definition of democracy, being the worst possible system except for all the alternatives. It is a system of metaphors and adjectives, that are capable of describing phenomena with some degree of inter-rater reliability, and the revised version has incorporated many of the advances in knowledge in the past decades regarding course, outcome and treatment response. It reflects the imperfection of the field, and needs to be used, as should all models, as something useful, acknowledging openly and consciously that it remains fundamentally wrong [4].

## Competing interests

Michael Berk has received Grant/Research Support from the NIH, Cooperative Research Centre, Simons Autism Foundation, Cancer Council of Victoria, Stanley Medical Research Foundation, MBF, NHMRC, Beyond Blue, Rotary Health, Geelong Medical Research Foundation, Bristol Myers Squibb, Eli Lilly, Glaxo SmithKline, Meat and Livestock Board, Organon, Novartis, Mayne Pharma, Servier and Woolworths, has been a speaker for Astra Zeneca, Bristol Myers Squibb, Eli Lilly, Glaxo SmithKline, Janssen Cilag, Lundbeck, Merck, Pfizer, Sanofi Synthelabo, Servier, Solvay and Wyeth, and served as a consultant to Astra Zeneca, Bristol Myers Squibb, Eli Lilly, Glaxo SmithKline, Janssen Cilag, Lundbeck Merck and Servier.

## Pre-publication history

The pre-publication history for this paper can be accessed here:

http://www.biomedcentral.com/1741-7015/11/128/prepub
